# Assessment of the Relationship between Selected Factors and Stress-Coping Strategies in Handcyclists—A Preliminary Study

**DOI:** 10.3390/medicina56050211

**Published:** 2020-04-27

**Authors:** Agnieszka Turoń-Skrzypińska, Wioletta Pawlukowska, Aleksandra Szylińska, Natalia Tomska, Anna Mikołajczyk-Kocięcka, Magdalena Ptak, Grażyna Dutkiewicz, Iwona Rotter

**Affiliations:** 1 Department of Medical Rehabilitation and Clinical Rehabilitation, Pomeranian Medical University in Szczecin, Żołnierska 54, 71-210 Szczecin, Poland; agi.skrzypinska@gmail.com (A.T.-S.); wsna@o2.pl (W.P.); aleksandra.szylinska@gmail.com (A.S.); mikolajczyk.ania1@wp.p (A.M.-K.); ptak.magda@gmail.com (M.P.); iwrot@wp.pl (I.R.); 2 Department of Nephrology, Transplantology and Internal Medicine, Pomeranian Medical University, 70-111 Szczecin, Poland; grazyna_dutkiewicz@o2.pl

**Keywords:** Paralympic sport, handbike, coping, stress, elite athletes, handcycling, disabled sport

## Abstract

*Background and Objectives*: Playing competitive sports is associated with stress, especially during the starting season. Disabled athletes are additionally burdened with physical and/or emotional factors, resulting from the trauma they have experienced. The aim of the work was to assess the relationship between strategies of coping with stress and the level of education, category of disability and its duration of handcyclists before the competition. *Materials and Methods*: 44 handcyclists with a mean age of 41.8 ± 11.6, from European countries, were divided according to the severity of mobility impairments, education and duration of the disability. The participants were asked to fill in the Mini-COPE Inventory for Measuring Coping with Stress, which provided answers in writing to some sociodemographic questions regarding age, sex, education, type of mobility impairment and duration of the disability. *Results:* The subjects who had suffered spinal injury at the cervical section obtained the lowest scores regarding their subjective assessment of their active stress management in difficult situations (*p* = 0.007). They scored the lowest, 1.5 points, when asked about acceptance in difficult circumstances compared to those with university education (*p* = 0.02). A statistically significant correlation was found to exist between education levels and positive revaluation, acceptance and seeking instrumental support. A negative correlation was observed between education and sustained use of psychoactive substances and denial. *Conclusions:* Highly educated cyclists with short-lasting disability, damage to the lower spine section or amputations tend to cope better with stress than other study participants.

## 1. Background

There are many definitions of stress that can be found in the literature. Hans Hugo Seyle, a Canadian physician, was the first to introduce the term “stress” or “degree of body wear” caused by physical and psychological factors. According to him, this term means “a nonspecific response of the body to any demand” [1]. The theory of coping with stress by Lazarus and Folkman, defines stress as a particular relationship between the person and the environment. The person interprets the situation as aggravating and negatively affecting his or her well-being [2]. Feelings of stress are experienced, among others, in traumatic and extraordinary situations, in relation to everyday life situations and while playing sports [3]. Stress has become part of everyday life and is counted in the group of diseases of the 21st century [4].

Reactions to stress may depend on the mental and physical state of the body, as well as on environmental, socio-cultural factors and the economic situation of an individual. The character of a person is the most important factor in determining the type of stress-induced reaction [5].

Stress factors can be short- and long-term and may vary in intensity. An example of a long-term factor is the presence of a chronic disease or disability. The occurrence of stressful situations can mobilize a person to action. However, as a result of prolonged stressful situations, disorganization and even destruction of the body can occur. Strong and prolonged stress is a negative phenomenon that leads to sleep disorders, hyperphagia, lack of appetite, excessive consumption of alcohol and cigarettes, and dysphasia. It leads to the deterioration of the efficiency and effectiveness of the functions of the body in various spheres of life [5]. In turn, coping with stress involves controlling the factors and situations that cause nervousness using protective and preventive skills [2].

Handcycling is a popular sport with people affected by a mobility impairment. As a Paralympic sport, a form of para-cycling, handcycling first appeared in Athens, Greece, in 2004 [6]. The rules and events were formulated by UCI (Union Cycliste Internationale). Depending on the function retained, the competitors belong to a given starting category. There are five starting categories: H1–H5, where H1 and H2 refer to cervical spine injuries and H3 means defects within the thoracic section. Groups H4 and H5 include contestants after lower limb amputations and injuries in the lumbar spine [7].

Playing competitive sports is associated with stress. The concept of stress in professional athletes has been widely described in the literature [8,9]. Studies show that different sources of stress can be distinguished in competitive sports. The stress factors can be unrelated to the sports played or directly connected with them [8,10]. It is believed that the main stress factors affecting athletes are related to their quality of life [11,12], job satisfaction [13], depression [14] and sleeping habits [2]. Other researchers claim that stress might be the result of a lack of balance between personal evaluation of one’s physical fitness and the actual performance [15]. Disabled athletes are additionally burdened with physical and/or emotional factors, resulting from the trauma they have experienced [16]. 

It is important to consider all the factors connected with stress affecting an athlete. There are, however, very few publications dealing with assessment of the influence of social factors and extent of a disability on the effectiveness of stress management [16], especially in handcyclists. 

The aim of the study was to assess the relationship between strategies of coping with stress and the level of education, category of disability and its duration in handcyclists before a competition.

## 2. Material and Methods

### 2.1. Characteristics of the Study Cohort

The study involved 44 disabled athletes from many European countries, 37 males (84%) and 7 females (16%), training for competitive handbiking, with a mean age of 41.8 ± 11.6 years. Six (14%) of the participants were from Poland, 19 (45%) from Germany, 2 (5%) came from Slovenia, 3 (7%) from Belgium, 3 (7%) from Switzerland, 5 (12%) from Austria and 3 (7%) from France. Of the entire group, 5 (12%) people completed a master’s degree course, 11 (25%) held a bachelor’s degree, 21 (47%) received a secondary education and the remaining 6 (14%) were vocational schools graduates.

The athletes were divided into 3 groups: 5 (12%) individuals with cervical spine defects, 15 (34%) suffering from injured spine within the thoracic section and 24 (54%) included those after lower limb amputations and injuries in the lumbar spine. The mean duration of impairment in all subjects was 19.3 ± 12.1. The disability was innate or acquired in 4 (9%) and 40 (91%) of the athletes, respectively (Table 1).

### 2.2. Methodology of the Study

The study was conducted on the day of the technical briefing before the international race of 2015 (Vuelta Playa Blanca, Lanzarote, Spain) and was preceded by obtaining the athletes’ written consent. The subjects were informed about the aim of the study and its anonymity. Subsequently, they were asked to answer some questions concerning their particulars, such as age, sex, education, type of mobility impairment and duration of the disability. Finally, they completed, in either Polish or English, the Mini-COPE Inventory for Measuring Coping with Stress. The questions in both language versions did not differ. The inventory contained 28 statements relating to 14 stress-coping strategies, which include active stress management, planning, positive revaluation, acceptance, sense of humour, faith, seeking emotional support, seeking instrumental support, getting engaged in various activities, denial, venting emotions, resignation and self-blame. Participants referred to the statements by circling options rising progressively from 0 to 3, where 0 means “I almost never do it” and 3 means “I almost always do it”. Each of the stress management strategies is evaluated separately. The higher the score the more frequently a given strategy is applied by the respondent [17].

The study was approved by the Pomeranian Medical University Commission of Ethics—Resolution no KB-0012/101/03/17, on 07.03.2016.

### 2.3. Statistical Analysis

Statistical analysis was made using elements of descriptive statistics, mainly means and standard deviations. The nonparametric Mann–Whitney U test was used for the assessment of the Mini-COPE Inventory in two categories of disability duration: one lasting over 15 years and the other lasting up to 15 years. The Kruskal–Wallis test was applied for the multi group evaluation of the level of education and sports category. Spearman’s rank correlation coefficient was used for the assessment of statistical dependence between the variables in the studied groups. Statistical significance was adopted at *p* < 0.05. Statistical analysis was performed using the Statistica 12 software package.

## 3. Results

The athletes competing were divided into different categories, which were determined by the section of spinal damage. Table 2 presents the athletes’ Mini-COPE scores divided according to sports categories. The subjects who had suffered spinal injury at the cervical section obtained the lowest scores regarding their subjective assessment of their active stress management in difficult situations (*p* = 0.007). They also received bottom scores regarding their positive revaluation (*p* = 0.02). Statistically significance was found between sense of humour and the athletes with thoracic section injury (*p* = 0.04).

Table 3 shows how the contestants’ Mini-COPE inventory scores relating to their education. The subjects with vocational education scored the lowest as for the question concerning positive revaluation (*p* = 0.05). In comparison to the subjects with a high level of education, they also received the lowest score (1.5) when answering the question concerning acceptance in difficult situations (*p* = 0.02).

Table 4 shows interdependencies between the Mini-COPE inventory scores and the separately analysed starting categories, levels of education and the length of disability. A statistically significant positive correlation was found between education, positive revaluation, acceptance and seeking instrumental support. A negative correlation was observed between education and taking psychoactive substances, getting engaged in various activities and denial. Participants’ starting categories correlated with active coping with stress, positive revaluation and sense of humour.

## 4. Discussion

There is no ideal way to manage stress [18]. Based on the classic approach to coping with stress proposed by Lazarus and Folkman, Carver et al. (1989) distinguished several strategies for coping with stress. They observed, among others, problem-focused strategies (e.g., active stress management and planning), remedial strategies focused on emotions (e.g., seeking emotional support) and avoidance behaviours, i.e., doing something else [19].

A review of the publication shows that different questionnaires and scales are used to assess stress management [20,21]. The culturally adapted coping scale (CSKA) was used in researching the stress-coping profile in a group of competitive athletes, depending on the performance and type of sport practiced [21]. In turn, the Coping Style in Sport Inventory (CSSI) was used in studies assessing the style of coping with stress by athletes studying at universities in Jordan [22]. The “Stress Coping Strategies Scale”, developed by Lazarus and Folkman (1984), was also used in the research aimed at assessing strategies of coping with stress in a group of athletes with disabilities playing amputee football, wheelchair basketball and swimming [2]. Carver et al. (1989) developed a stress management instrument, which they called COPE [19], and then Carver (1997) developed a short version of this instrument called Brief COPE [23]. In Poland, the adaptation is used, which is called Mini-COPE, which was used in this study [17]. In the research on strategies of coping with stress in a group of Portuguese athletes practicing group and individual sports, the same inventory of coping with stress, a Portuguese adaptation, has been used [24].

Sport for people with disabilities is of great importance for supporting social, physical and mental development, as well as for improving their overall quality of life [25,26,27]. There are many sports adapted to the needs of people with disabilities. One of them is handcycling, which is a combination of a wheelchair and a bicycle [28]. The accessibility of the world for people limited to a wheelchair has been increased, and it has been more widely opened to sport [29,30,31,32].

The studies published so far have shown that the physiological and psychosocial response to stress is less intense in people who train regularly, recreationally and competitively [4]. Participation in sport and an element of competition develops a sense of belonging to a group and allows a person to accept physical disability more easily. At a master level, for people with disabilities, sport has become more and more competitive. Just as in sport for people without any disabilities, there are successes, disappointments and failures. Extreme demands are placed on athletes, which can cause stressful situations [33].

There are many publications in the literature on the use of various strategies of coping with stress in sport for people without disabilities [33]. Among other things, the impact that styles of coping with stress has on the sport performance in athletes studying at the University of Tehran was assessed [34]. Stress-coping styles were also analysed in a group of men and women playing football [35,36,37], volleyball [38], table tennis players [39], in MMA players [40] and golf players [41].

However, the available literature still has a limited number of studies on strategies of coping with stress for athletes with disabilities [5]. Our own research has shown that their level of coping with stress is related to the level of spinal cord injury, education and duration of disability. This results in a conclusion that, among others, anatomical and social conditions are important factors in coping with stress. In our research, people with cervical spine injury had higher levels of stress. However, in the study of Flyn et al. (2011) on the attitudes of people with tetraplegia and paraplegia, it was found that people with paraplegia have a better attitude than people with tetraplegia [42]. The study of Eraslan et al. (2017) also showed that the perception of stress by people with disabilities depends, among other things, on the degree of the disability, which was also confirmed in our studies. Better physical fitness positively affects the ability to deal with stress in athletes with disabilities. People with higher education and a shorter duration of disability deal with stress more easily. Previous studies have shown that people with a longer duration of disability have more physical and psychological problems. Other studies have shown that people with a longer duration of disability have poor integration with society [5]. In the athletes participating in our study, the same factors could also have contributed to a higher level of stress than in people with a shorter duration of disability.

Earlier studies in athletes with spinal cord injuries (SCI) focused on the analysis of strength, ergometry and mental well-being, showing that long-term and short-term physical training, improving physical and mental performance, is possible. When it comes to Paralympians, research focused on the study of mechanical performance, the effect of training upper body muscle endurance and the effects of interval training [43]. Havva (2019), in turn, conducted research of differences in fear of social appearance and self-esteem in athletes with and without disabilities. He showed that disabled people had a higher level of anxiety associated with social appearance compared to athletes without disabilities [44].

In the literature it is also possible to find research on the relationship between strategies of coping with stress and acceptance of chronic illness using Mini-Cope [45]. Research conducted by Zaher et al. (2010) proves that the occurrence of a chronic disease significantly affects the choice of strategies of coping with stress [46]. It has been shown that respondents in stressful situations more often turn to religion [45]. The use of different strategies of coping with stress can be influenced by character traits, the cultural environment or level of education [47], which was also described in our research. People with high self-esteem, positive thinking and low neuroticism are characterized by greater psychological endurance and more effective mechanisms of coping with stress [48]. Based on the available research, it can also be stated that the amount of time playing sports is a factor directly affecting the strategy of coping with stress [5]. People who practice sport regularly cope with stress better compared to people who do not play any sport [49].

Available studies have shown that ways of coping with stress can be related to experience gained and the level of sport training [50]. It has been proven that athletes practicing team sports, compared to people from the general population, more often show a task-orientated style, while less often a style focused on avoidance. Martial arts athletes less often use emotion-oriented and avoidance-oriented styles to deal with stress [51].

No similar research on a group of paracyclists, including handcyclists, has been found in the literature. Therefore, the research conducted by us is valuable. It is an excellent starting point for conducting a prospective study on a larger group of athletes.

### Limitations

The main limitation of the study was a relatively small number of athletes training for handcycling. Yet, it is worth stressing that, as the sport is rapidly gaining popularity among people with disabilities, prospective study participant pools are continually expanding. Thus, it seems that further research should be recommended.

## 5. Conclusions

Cyclists with injuries to the lumbar section of the spinal cord and lower limb amputations demonstrate higher levels of ability to cope with stress, pressure and strong emotions. They are more likely to mitigate the harmful effects of negative emotions with humour and a positive attitude. Athletes that are highly educated present much better acceptance and positive revaluation in stress situations. Long lasting disability tends to lead to helplessness, resignation and a lack of efforts in highly stressful situations. Long-term disability leads to resignation in extremely stressful situations.

## Figures and Tables

**Table 1:** Group characteristics.

**Sex**	Male (*n*, %)	37 (84%)
	Female (*n*, %)	7 (16%)
Age (AM ± SD)		41.8 ± 11.6
**Participants (*n*, %)**	Poland	6 (14%)
	Germany	19 (45%)
	Slovenia	2 (5%)
	Belgium	3 (7%)
	Switzerland	3 (7%)
	Austria	5 (12%)
	France	3 (7%)
**Education (*n*, %)**	Master’s degree	5 (12%)
	Bachelor’s degree	11 (25%)
	Secondary education	21 (47%)
	Vocational school graduates	6 (14%)
**Spine injury (*n*, %)**	Cervical section	5 (12%)
	Thoracic section	15 (34%)
	Lumbar section	24 (54%)
Duration of impairment in all subjects (AM ± SD)		19.3 ± 12.1
**Disability (*n*, %)**	Innate	4 (9%)
	Acquired	40 (91%)

*n*—number of patients, %—value in percent, AM—average, SD—standard deviation.

**Table 2:** Analysis the average scores of the Mini-COPE inventory for various starting categories.

Methods of Coping with Stress	Participants Starting Categories						
	Cervical Section Injury (*n* = 5)		Thoracic Section Injury (*n* = 15)		Lumbar Section Injury and Lower Limbs Amputation (*n* = 24)		*p* Value
	M ± SD	Me	M ± SD	Me	M ± SD	Me	
Active coping with stress	1.00 ± 0.35	1.0	2.07 ± 0.59	2.0	2.08 ± 0.82	2.0	0.007 *
Planning	1.40 ± 0.65	1.0	1.90 ± 0.78	2.0	1.96 ± 0.76	2.0	0.28
Positive revaluation	1.00 ± 0.35	1.0	1.77 ± 0.67	2.0	2.00 ± 0.83	2.0	0.02 *
Acceptance	1.80 ± 0.91	2.0	2.17 ± 0.65	2.0	2.44 ± 0.65	2.5	0.19
Sense of humour	1.80 ± 1.09	1.0	1.40 ± 0.85	1.0	2.15 ± 0.84	2.0	0.04 *
Turning to religion/meditation	0.30 ± 0.67	0.0	0.50 ± 0.93	0.0	0.42 ± 0.79	0.0	0.54
Seeking emotional support	1.70 ± 0.76	1.5	1.67 ± 0.79	1.5	1.75 ± 0.73	2.0	0.24
Seeking instrumental support	1.60 ± 0.55	1.5	1.73 ± 0.84	2.0	1.79 ± 0.75	2.0	0.74
Getting engaged in various activities	2.00 ± 0.61	2.0	1.57 ± 0.73	1.5	1.65 ± 0.79	1.5	0.51
Denial	0.90 ± 0.89	1.0	0.87 ± 0.81	1.0	0.79 ± 0.81	0.8	0.85
Venting emotions	1.60 ± 0.42	1.5	1.17 ± 0.65	1.0	1.31 ± 0.70	1.3	0.69
Taking psychoactive substances	0.50 ± 0.61	0.5	0.77 ± 0.92	0.5	0.38 ± 0.69	0.0	0.51
Resignation	0.80 ± 0.76	1.0	0.93 ± 0.75	1.0	0.77 ± 0.71	1.0	0.75
Self-blame	1.00 ± 0.50	1.0	1.00 ± 0.93	1.0	1.31 ± 0.78	1.3	0.64

*n*—number of patients, M—average, SD—standard deviation, Me—median, *p*-value—statistical significance *.

**Table 3:** Mean Mini-COPE inventory scores according to level of education.

Strategies for Coping with Stress	Education								
	Vocational (*n* = 6)		Secondary (*n* = 21)		Bachelor’s Degree (*n* = 11)		Master’s Degree (*n* = 6)		*p* Value
	M ± SD	Me	M ± SD	Me	M ± SD	Me	M ± SD	Me	
Active coping with stress	1.42 ± 0.58	1.3	2.02 ± 0.77	2.0	1.95 ± 0.96	2.0	2.25 ± 0.42	2.0	0.15
Planning	1.25 ± 0.82	1.3	1.90 ± 0.77	20	2.00 ± 0.55	2.0	2.17 ± 0.88	2.5	0.19
Positive revaluation	0.92 ± 0.86	0.8	1.88 ± 0.67	2.0	2.14 ± 0.67	2.0	1.83 ± 0.88	1.8	0.05 *
Acceptance	1.50 ± 0.45	1.5	2.26 ± 0.57	2.5	2.55 ± 0.57	3.0	2.58 ± 0.58	2.8	0.02 *
Sense of humour	1.67 ± 1.08	1.8	1.67 ± 0.82	1.5	2.36 ± 0.89	3.0	1.75 ± 0.98	2.0	0.17
Turning to religion/meditation	0.58 ± 0.74	0.3	0.21 ± 0.51	0.0	0.41 ± 0.73	0.0	1.08 ± 1.49	0.3	0.27
Seeking emotional support	1.67 ± 0.68	1.8	1.55 ± 0.79	1.5	1.91 ± 0.54	2.0	2.00 ± 0.95	2.0	0.41
Seeking instrumental support	1.17 ± 0.61	1.3	1.74 ± 0.70	2.0	1.95 ± 0.72	2.0	2.00 ± 0.95	2.0	0.16
Getting engaged in various activities	2.08 ± 0.58	2.3	1.76 ± 0.64	1.5	1.36 ± 0.87	1.0	1.42 ± 0.86	1.5	0.16
Denial	1.58 ± 1.02	1.8	0.85 ± 0.78	1.0	0.59 ± 0.54	0.5	0.42 ± 0.66	0.0	0.09
Venting emotions	1.92 ± 0.66	2.0	1.21 ± 0.60	1.0	1.18 ± 0.72	1.0	1.17 ± 0.52	1.0	0.15
Taking psychoactive substances	1.17 ± 1.13	0.8	0.60 ± 0.82	0.0	0.27 ± 0.41	0.0	0.08 ± 0.20	0.0	0.07
Resignation	0.83 ± 0.68	1.0	1.00 ± 0.76	1.0	0.86 ± 0.64	1.0	0.17 ± 0.41	0.0	0.08
Self-blame	1.00 ± 0.99	1.0	1.26 ± 0.83	1.5	1.41 ± 0.70	1.5	0.58 ± 0.49	0.8	0.15

*n*—number of patients, M—average, SD—standard deviation, Me—median *p*-value—statistical significance *.

**Table 4:** Analysis of the interdependencies between the Mini-COPE inventory scores, starting categories, levels of education and the length of disability.

Strategies for Coping with Stress	Participants Starting Categories (*n* = 44)		Education (*n* = 44)		Length of Disability (*n* = 44)	
	R Spearman	*p* Value	R Spearman	*p* Value	R Spearman	*p* Value
Active coping with stress	0.335	0.026 *	0.335	0.026 *	−0.081	0.601
Planning	0.170	0.270	0.170	0.270	−0.017	0.915
Positive revaluation	0.355	0.018 *	0.355	0.018 *	−0.104	0.500
Acceptance	0.275	0.071	0.275	0.071	−0.132	0.392
Sense of humour	0.323	0.032 *	0.323	0.032 *	−0.256	0.094
Turning to religion/meditation	0.007	0.0963	0.007	0.0963	0.279	0.067
Seeking emotional support	0.065	0.676	0.065	0.676	0.020	0.896
Seeking instrumental support	0.110	0.477	0.110	0.477	0.072	0.642
Getting engaged in various activities	−0.088	0.568	−0.088	0.568	0.167	0.279
Denial	−0.051	0.741	−0.051	0.741	0.115	0.456
Venting emotions	−0.056	0.718	−0.056	0.718	−0.026	0.868
Taking psychoactive substances	−0.208	0.175	−0.208	0.175	−0.036	0.819
Resignation	−0.071	0.645	−0.071	0.645	0.389	0.011
self-blame	0.174	0.260	0.174	0.260	0.048	0.758

*n*—number of patients, *p*-value—statistical significance *.
